# The Effect of Protocatechuic Acid on Blood Pressure and Oxidative Stress in Glucocorticoid-induced Hypertension in Rat

**Published:** 2016

**Authors:** Leila Safaeian, Valiollah Hajhashemi, Shaghayegh Haghjoo Javanmard, Hamed Sanaye Naderi

**Affiliations:** a *Department of Pharmacology and Toxicology and Isfahan Pharmaceutical Sciences Research Center, School of Pharmacy and Pharmaceutical Sciences, Isfahan University of Medical Sciences, Isfahan, Iran. *; b *Applied Physiology Research Center, Isfahan University of Medical Sciences, Hezar Jarib Avenue, Isfahan, Iran.*

**Keywords:** Protocatechuic acid, Hypertension, Dexamethasone, Antioxidants

## Abstract

Oxidative stress is one of the important mechanisms involved in Dexamethasone (Dex)-induced hypertension. Protocatechuic acid (PCA) is a natural compound with high antioxidant capacity. In this investigation, the effect of pretreatment with PCA was studied in Dex-induced hypertensive male Wistar rats. For induction of hypertension, Dex was injected subcutaneously for 14 days. PCA (50, 100 and 200 mg/kg) was started from 4 days before Dex administration and continued during the test period. Systolic blood pressure (SBP) was recorded using tail-cuff method. Measurement of thymus weight was done as a marker of glucocorticoid activity. The hydrogen peroxide (H_2_O_2_) concentration and ferric reducing antioxidant power (FRAP) were determined in plasma samples. Significant increase in SBP and plasma H_2_O_2 _concentration and decrease in FRAP value and in the body and thymus weights were observed in Dex-induced hypertensive rats. PCA dose-dependently prevented hypertension and body weight loss, and reduced plasma H_2_O_2 _concentration and increased FRAP values. These results suggest the antihypertensive and antioxidant effects of PCA against Dex-induced hypertension.

## Introduction

Hypertension (HTN) is a common medical condition which approximately affects 40% of people in the world (1). HTN is a risk factor for many cardiovascular diseases such as angina, congestive heart failure, coronary heart disease (CHD), increased intraocular pressure, retinal microvascular abnormalities, cerebrovascular accidents, myocardial infarction (MI) and end-stage renal disease (2, 3). As a main cause of death and disability, HTN is a major concern for worldwide health (4).

A growing body of evidence suggests that oxidative stress plays a great role in the pathogenesis of hypertension (5). Increased production of reactive oxygen species (ROS) such as superoxide anion radicals, hydroxyl radicals, hydrogen peroxide and singlet oxygen occurs in oxidative status (6). Interaction between ROS and nitric oxide (NO) which leads to decrease NO availability and imbalance between free radical production and antioxidant defense have been implicated in the pathophysiology of vasoconstriction and high blood pressure (7). Some beneficial effects have been reported from antioxidant agents in the prevention and attenuation of hypertension and also improvement of endothelial dysfunction (8, 9).

Protocatechuic acid (PCA) is a natural phenolic compound and major metabolite of anthocyanins with free radical scavenging action (10, 11). It has various pharmacological activities including anti-inflammatory, anti-apoptotic, anti-diabetic and neuroprotective effects (12-14). PCA also possesses beneficial cardiovascular properties such as anti-atherogenic and endothelial cell-protective effects (15, 16). Since the anti-hypertensive activity of PCA has remained unknown, the present study was designed to evaluate the effects of chronic administration of PCA on dexamethasone (Dex)-induced hypertension in rats. 

## Experimental

*Animals*

Male albino Wistar rats (200 ± 20 g) were obtained from the animal house of the School of Pharmacy and Pharmaceutical Sciences, Isfahan University of Medical Sciences, Iran. They were housed under standard the laboratory conditions and had access to standard rat chow and water. Rats were allowed to acclimatize to laboratory condition for 1 week before the experiments. All the experimental procedures were according the standard ethical guidelines for laboratory animal use and care as described by the Animal Ethical Committee of Isfahan University of Medical Sciences.

*Materials*

Protocatechuic acid (3, 4-dihydroxybenzoic acid) was obtained from Serva Feinbiochemica (Heidelberg, Germany). Dexamethasone and vitamin C (ascorbic acid) were purchased from Darou Pakhsh Pharmaceutical Co. (Tehran, Iran) and captopril was purchased from Tehran Darou Pharmaceutical Co. (Tehran, Iran). The measurement of plasma lipid hydroperoxides and ferric reducing antioxidant power (FRAP) were done using standard assay kits (Hakiman Shargh Research Co., Isfahan, Iran). 

*Experimental protocol*

In this prevention study, oral administration of drugs using an intragastric tube was started from 4 days before induction of hypertension by Dex and continued during the test period. Rats were randomly divided into seven groups as follows: (i) group 1 as the saline control group received daily injection of saline (1 mL/kg, s.c.) for 14 days (Days 4-18); (ii) group 2 as the Dex control group received daily injection of Dex (30 µg/kg, s.c.) for 14 days (Days 4-18) (17); (iii) group 3 as the antihypertensive positive control group received daily oral administration of captopril (40 mg/kg) (Days 0-18); (iv) group 4 as the antioxidant positive control group received daily oral administration of vitamin C (750 mg/kg) (Days 0-18); groups 5 to 7 received daily oral administration of different doses of PCA (50, 100 and 200 mg/kg) (Days 0-18).

The saline and Dex control rats were also received PCA vehicle (1% carboxymethyl cellulose in saline) orally. Six rats were used in each group. Rats were weighed on alternate days between 10 AM and 12 noon. At the end of the experiment, all groups of animal were sacrificed under ether anesthesia. The thymus gland was removed. The blood samples were collected into heparinized tubes and plasma was separated for further experiments. 

*Measurement of systolic blood pressure*

The systolic blood pressure (SBP) was recorded at the first day and the last day of the experiment (days 1 and 18), between 10 AM and 12 noon, by non-invasive tail-cuff method (AD Instrument PowerLab Data Acquisition System, Australia). Rats were trained with instrument for one week before initiation of the experiment and were conscious during the measurement. The animals were placed in a heated restrainer at 37 ± 1 ºC for 10 minutes. At least 3 blood pressures were measured for each rat and the average of them was reported as the SBP. 

*Measurement of thymus weight*

The thymus weight, as a marker of glucocorticoid activity was measured and reported as mg/100g of body weight (18). 

*Measurement of plasma hydrogen peroxide concentration*

A colorimetric assay kit based on the ferrous ion oxidation by xylenol orange reagent in aqueous medium with sorbitol (FOX1) was used for the measurement of plasma hydrogen peroxide (H_2_O_2_) concentration (19). In brief, FOX1 reagent prepared according to the manufacturer’s protocol was mixed with the plasma samples and incubated for 30 min in 37 ºC. The absorbance of solutions was measured at 540 nm using a microplate reader/spectrophotometer (Bio-Tek, Power Wave XS). The H_2_O_2_ concentration of plasma samples was calculated using a standard curve obtained from different concentrations of H_2_O_2_.

*Measurement of plasma ferric reducing antioxidant power*

A colorimetric assay kit was used for measurement of the total antioxidant capacity of plasma samples by ferric reducing antioxidant power (FRAP) assessment (20). FRAP was evaluated based on the reduction of ferric-tripyridyltriazine complex to ferrous form. In brief, the FRAP reagent according to the manufacturer’s protocol was added to the plasma samples and incubated for 40 min in 40 °C. The absorbance of colored solutions was measured at 570 nm using a microplate reader/spectrophotometer. The FRAP value of samples were calculated against the standard curve of FeSO4x7H2O concentrations and expressed as micromole of Fe II equivalents per liter.

*Statistical analysis*

Statistical analysis was performed by one-way analysis of variance (ANOVA) followed by Tukey post-hoc test using SPSS software version 16.0. The data were shown as the mean ±SEM. *P *value* < *0*.*05 was considered to be statistically significant.

## Results

*Effect of protocatechuic acid* *on blood pressure*

**Figure 1:**
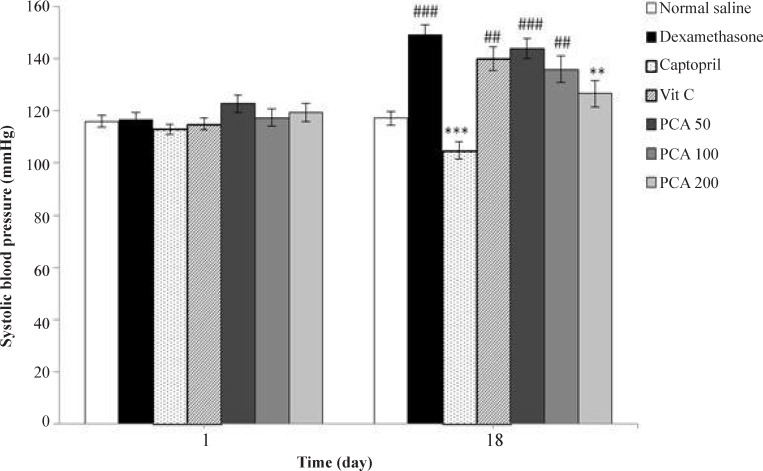
Effects of pretreatment with PCA (50, 100 and 200 mg/kg), captopril (40 mg/kg) and vitamin C (750 mg/kg) on systolic blood pressure in Dex-induced hypertension. Values are means + SEM for six rats. ^**^P < 0.01, ^***^P < 0.001 as compared to Dex control group. ^##^*P* < 0.01 and ^###^*P* < 0.001 as compared to saline control group

 Daily injection of dexamethasone significantly increased SBP from 116.8 + 2.5 to 149.2 + 3.9 mmHg on day 18 (*P* < 0.001) in comparison with the saline control group (117.2 + 2.6 mmHg). Figure 1 shows the effect of pretreatment with PCA (50, 100 and 200 mg/kg), captopril (40 mg/kg) and vitamin C (750 mg/kg) on SBP in Dex-induced hypertension. Pretreatment with PCA at a dose of 200 mg/kg significantly prevented the Dex-induced hypertension in rats (*P* < 0.01). However vitamin C had no effect and captopril had more potent effect than PCA on prevention of hypertension (*P* < 0.001).

*Effect of protocatechuic acid* *on body weight*

**Figure 2.**
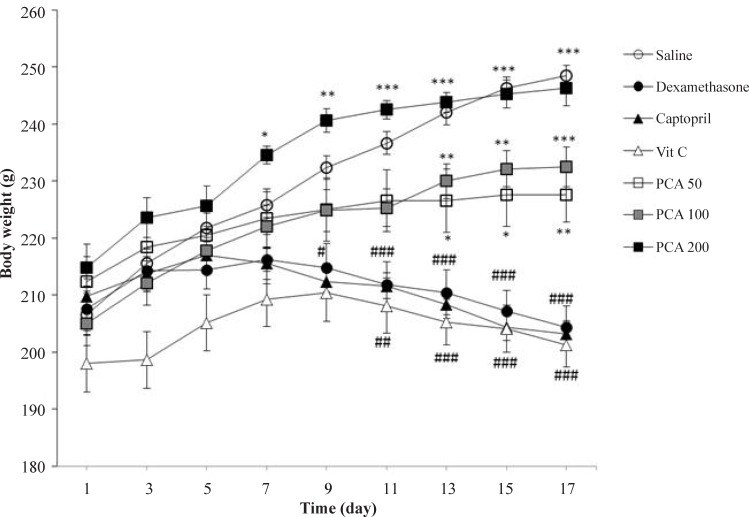
Effects of pretreatment with PCA (50, 100 and 200 mg/kg), captopril (40 mg/kg) and vitamin C (750 mg/kg) on body weight in Dex-induced hypertension. Values are means + SEM for six rats. ^*^P < 0.05, ^**^P < 0.01 and ^***^P < 0.001 as compared to Dex control group. ^#^*P* < 0.05, ^##^*P* < 0.01 and ^###^*P* < 0.001 as compared to saline control group

The body weight significantly decreased in Dex-induced hypertensive rats when compared to saline control group (*P* < 0.001). PCA administration improved weight gaining in rats at all doses but captopril and vitamin C could not prevent from the weight loss induced by dexamethasone (Figure 2).

*Effect of protocatechuic acid* *on thymus weight*

**Figure 3:**
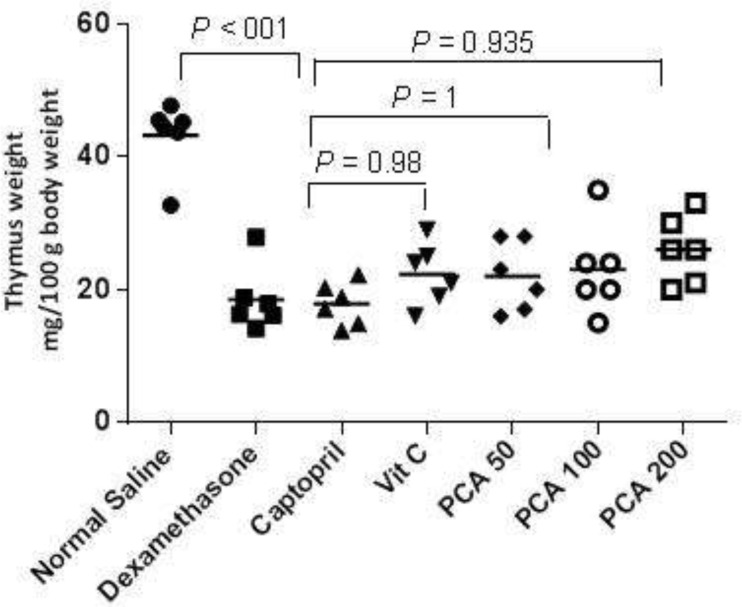
Effects of pretreatment with PCA (200 mg/kg), captopril (40 mg/kg) and vitamin C (750 mg/kg) on thymus weight in Dex-induced hypertension. Values are means for six rats. *P* < 0.05 is considered to be statistically significant versus Dex control group

The Dex treatment significantly decreased the thymus gland weight (*P* < 0.001) but pretreatment with PCA, captopril and vitamin C could not prevent the thymus weight decrease (Figure 3).

*Effect of protocatechuic acid* *on plasma *H_2_O_2_* concentration*

**Figure 4:**
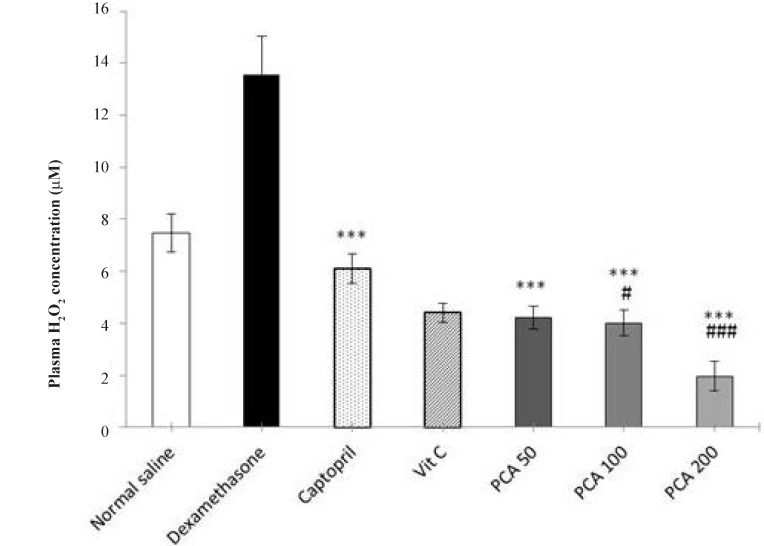
Effects of pretreatment with PCA (50, 100 and 200 mg/kg), captopril (40 mg/kg) and vitamin C (750 mg/kg) on plasma H_2_O_2_ concentration in Dex-induced hypertension. Values are means + SEM for six rats. ^***^P < 0.001 as compared to Dex control group. ^#^*P* < 0.05 and ^###^*P* < 0.001 as compared to saline control group

The level of plasma H_2_O_2_ in Dex-treated group was significantly higher than the saline control group (*P* < 0.001). Pretreatment with PCA, captopril and vitamin C significantly prevented the rise in plasma H_2_O_2_ concentration (*P* < 0.001). At doses of 100 and 200 mg/kg of PCA, the plasma H_2_O_2_ concentration was also significantly lower than the saline control group (Figure 4). 

*Effect of protocatechuic acid* *on plasma* *FRAP* *value*

**Figure 5:**
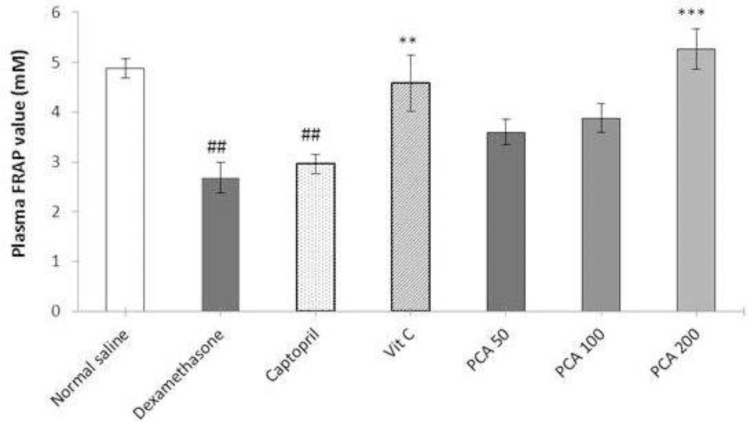
Effects of pretreatment with PCA (50, 100 and 200 mg/kg), captopril (40 mg/kg) and vitamin C (750 mg/kg) on plasma FRAP value in Dex-induced hypertension. Values are means + SEM for six rats. ^**^P < 0.01 and ^***^P < 0.001 as compared to Dex control group. ^##^*P* < 0.01 as compared to saline control group

There was significant reduction in the plasma FRAP value in Dex-induced hypertensive rats compared with the saline control rats (*P* < 0.001). Pretreatment with vitamin C and PCA significantly prevented the decline in plasma FRAP values. Captopril pretreatment had no significant effect on the FRAP value (Figure 5).

## Discussion

In this study, pretreatment with protocatechuic acid at a dose of 200 mg/kg reduced SBP in Dex-induced hypertension in rats. Moreover, PCA reduced the plasma H_2_O_2_ concentration and improved the total antioxidant capacity of plasma. Chronic use of dexamethasone, the most potent synthetic glucocorticoid leads to hypertension (21). Animal model of Dex-induced hypertension allows investigating the effect of various natural and synthetic compounds on high blood pressure. The mechanisms by which glucocorticoids induce hypertension may result from increased activity of vasoconstrictor system including renin-angiotensin, endothelin and sympathetic system, deficiency in vasodilators such as NO, increased oxidative stress and hemodynamic alterations (21-22). Accumulated evidence has shown the important role of oxidative stress in Dex-induced elevated blood pressure. Dex administration is associated with suppression of anti-oxidants such as superoxide dismutase, over-production of ROS in vasculature through NAPDH oxidase pathway or possibly cyclooxygenase pathway and increased NO removal by oxidative stress which leads to vasoconstriction (6, 22, 23). In the current study, we observed an increase in the level of H_2_O_2_ and a decrease in the ferric reducing antioxidant power in the plasma of Dex-induced hypertensive rats.

PCA is a natural compound with nutritional value because of high daily intake of anthocyanins in comparison with other polyphenols (11). Anthocyanins which are found at great concentrations in vegetables and fruits have been widely considered for their health-promoting properties especially lowering the risk of cardiovascular diseases (24). PCA has a beneficial role in human diseases prevention because of various biological activities (11). Some helpful effects of PCA on cardiovascular system have been reported previously. PCA has anti-atherogenic and endothelial cell-protective effects partly due to its anti-inflammatory activity through inhibition of vascular cell adhesion molecule 1 (VCAM-1) and intercellular adhesion molecule 1 (ICAM-1) expression and reduction of NF-κB activity (15, 16). It also possesses inhibitory effects on platelet activation and low-density lipoprotein (LDL) oxidation (25, 26).

The antihypertensive effects of PCA may be attributed to its NADPH oxidase inhibition activity in endothelial cells (27). NADPH oxidase acts as a main producer of vascular ROS especially upon Dex-induced hypertension (22). 

During hypertension, rapidly interaction between excess ROS and NO inactivates NO and leads to formation of powerful oxidant, peroxynitrite resulting in a state of NO deficit (22). The antioxidant activities of PCA are created by both chelating metal transition ions and by scavenging free radicals via donating hydrogen atom or electron (28). Therefore antioxidant supplementation may be contributed in improving NO availability and endothelial function in hypertension.

The role of endothelial cyclooxygenase (COX) in superoxide overproduction during Dex-induced hypertension has not been fully studied. Endothelial COX has a role in the regulation of vascular tone. Arachidonic acid metabolism by endothelial COX is a source of superoxide production and COX contributes to the reduction of nitric oxide availability (29). It has been reported that PCA possesses the ability to suppress COX-2 activity and also to decrease COX-2 expression in some tissues and this fact may also be involved in its helpful effect in the prevention of hypertension (13).

The results of this study showed no antihypertensive effects for vitamin C supplementation in Dex-induced hypertension in spite of its antioxidant effects on reduction of plasma H_2_O_2_ concentration and improvement of plasma total antioxidant capacity. In the study of Akpaffiong *et al*. ascorbic acid administration could decrease blood pressure in spontaneously hypertensive rats (SHR) without significant effect on blood pressure in Wistar-Kyoto (WKY) rats (30). In another study, dietary supplementation with high doses of vitamin C (1000 mg/kg) for a long test period (9 weeks) has lowered the SBP in SHR rats (31). Variation in the doses and duration of treatment between studies and differences in the pathophysiological mechanisms involved in the various models of hypertension may be involved in different effect of vitamin C on blood pressure. Further studies are still needed for understanding the detail mechanisms of antihypertensive effect of PCA and also vitamin C in different models of hypertension.

A large body of evidence has confirmed involvement of the hypothalamus–pituitary–adrenal axis in the regulation of body weight. It has been found that corticotropin-releasing hormone (CRH) lowers the body weight set point. In some animal studies, administration of glucocorticoids at high doses has lowered the body weight set point. This effect may be due to the interactions of glucocorticoids with leptin. Glucocorticoids at supra-physiologic levels stimulate leptin production and secretion. Leptin may act as a signal reflecting the status of fat stores into the central nervous system. It also increases hypothalamic CRH production. Moreover, increasing the lipoprotein lipase activity and a rise in lipolyse has been observed during administration of high doses of glucocorticoids (32, 33). In this study, pretreatment with PCA at all doses could prevent the effect of Dex on body weight in rats.

The thymus gland weight is a well-known marker of glucocorticoid activity which reflects the apoptotic effects of glucocorticoids on thymocytes and lymphocytes (7). The thymus as a key organ in T lymphocyte ontogenesis has an important role in optimizing immune system function throughout the life (34). The results of investigations have shown that despite the severe deleterious effects on thymus gland, thymopoiesis could be restored to the normal levels after discontinuation of Dex treatment (35). Some treatments have been also able to prevent thymocyte apoptosis induced by Dex administration (34). However in the present study, pretreatment with all drugs could not prevent the thymus weight loss.

## Conclusion

In conclusion, these findings suggest that supplementation with PCA as a natural compound with health promoting actions might be helpful for the prevention of hypertension through reduction of blood pressure and improvement of oxidative status.

## References

[1] Feng XL, Pang M, Beard J (2014). Health system strengthening and hypertension awareness, treatment and control: data from the China Health and Retirement Longitudinal Study. Bull. World Health Organ.

[2] Wang S, Xu L, Jonas JB, Wong TY, Cui T, Li Y, Wang YX, You QS, Yang H, Sun C (2009). Major eye diseases and risk factors associated with systemic hypertension in an adult Chinese population: the Beijing Eye Study. Ophthalmology.

[3] Maki KC, Rains TM, Schild AL, Dicklin MR, Park KM, Lawless AL, Kelley KM (2013). Effects of low-fat dairy intake on blood pressure, endothelial function, and lipoprotein lipids in subjects with prehypertension or stage 1 hypertension. Vasc. Health Risk Manag.

[4] Meshram II, Arlappa N, Balkrishna N, Rao KM, Laxmaiah A, Brahmam GN (2012). Prevalence of hypertension, its correlates and awareness among adult tribal population of Kerala state, India. J. Postgrad. Med.

[5] Safaeian L, Ghasemi-Dehkordi N, Javanmard SH, Namvar H (2015). Antihypertensive and antioxidant effects of a hydroalcoholic extract obtained from aerial parts of Otostegia persica. (Burm) Boiss. Res. Pharm. Sci.

[6] Güder A, Korkmaz H (2012). Evaluation of in-vitro antioxidant properties of hydroalcoholic solution extracts Urtica dioica L, Malva neglecta Wallr and their mixture. Iran. J. Pharm. Res.

[7] Safaeian L, Zabolian H (2014). Antioxidant effects of bovine lactoferrin on dexamethasone-induced hypertension in rat. ISRN Pharmacol.

[8] Zhang Y, Croft KD, Mori TA, Schyvens CG, McKenzie KU, Whitworth JA (2004). The antioxidant tempol prevents and partially reverses dexamethasone-induced hypertension in the rat. Am. J. Hypertens.

[9] Houston MC (2013). The role of nutrition, nutraceuticals, vitamins, antioxidants, and minerals in the prevention and treatment of hypertension. Altern. Ther. Health Med.

[10] An LJ, Guan S, Shi GF, Bao YM, Duan YL, Jiang B (2010). Antihyperglycemic effect of protocatechuic acid on streptozotocin-diabetic rats. J. Basic Clin. Physiol. Pharmacol.

[11] Masella R, Santangelo C, D'Archivio M, Li Volti G, Giovannini C, Galvano F (2012). Protocatechuic acid and human disease prevention: biological activities and molecular mechanisms. Curr. Med. Chem.

[12] Deng JS, Lee SD, Kuo WW, Fan MJ, Lin YM, Hu WS, Huang YC, Velmurugan BK, Tsai FJ, Tsai CH, Huang CY (2014). Anti-apoptotic and pro-survival effect of protocatechuic acid on hypertensive hearts. Chem. Biol. Interact.

[13] Tsai SJ, Yin MC (2012). Anti-glycative and anti-inflammatory effects of protocatechuic acid in brain of mice treated by D-galactos. Food Chem. Toxicol.

[14] Harini R, Pugalendi KV (2010). Antihyperglycemic effect of protocatechuic acid on streptozotocin-diabetic rats. J. Basic Clin. Physiol. Pharmacol.

[15] Wang D, Wei X, Yan X, Jin T, Ling W (2010). Protocatechuic acid, a metabolite of anthocyanins, inhibits monocyte adhesion and reduces atherosclerosis in apolipoprotein E-deficient mice. J. Agric. Food. Chem.

[16] Zhou-Stache J, Buettner R, Artmann G, Mittermayer C, Bosserhoff AK (2002). Inhibition of TNF-alpha induced cell death in human umbilical vein endothelial cells and Jurkat cells by protocatechuic acid. Med. Biol. Eng. Comput.

[17] Zhang Y, Wu JH, Vickers JJ, Ong SL, Temple SE, Mori TA, Croft KD, Whitworth JA (2009). The role of 20-hydroxyeicosatetraenoic acid in adrenocorticotrophic hormone and dexamethasone-induced hypertension. J. Hypertens.

[18] Ong SL, Vickers JJ, Zhang Y, McKenzie KU, Walsh CE, Whitworth JA (2007). Role of xanthine oxidase in dexamethasone-induced hypertension in rats. Clin. Exp. Pharmacol. Physiol.

[19] Wolf SP (1994). Ferrous ion oxidation in presence of ferric ion indicator xylenol orange for measurement of hydroperoxides. Methods Enzymol.

[20] Benzie IF, Strain JJ (1996). The ferric reducing ability of plasma (FRAP) as a measure of “antioxidant power”: the FRAP assay. Anal. Biochem.

[21] Ong SL, Whitworth JA (2011). How do glucocorticoids cause hypertension: role of nitric oxide deficiency, oxidative stress, and eicosanoids. Endocrinol. Metab. Clin. North. Am.

[22] Ong SLH, Zhang Y, Whitworth JA (2009). Mechanisms of Dexamethasone-Induced Hypertension. Curr. Hypertens. Rev.

[23] Mondo CK, Yang WS, Zhang N, Huang TG (2006). Anti-oxidant effects of atorvastatin in dexamethasone-induced hypertension in the rat. Clin. Exp. Pharmacol. Physiol.

[24] Wallace TC (2011). Anthocyanins in cardiovascular disease. Adv.Nutr.

[25] Chang WC, Hsu FL (1992). Inhibition of platelet activation and endothelial cell injury by polyphenolic compounds isolated from lonicera japonica thunb Prostaglandins Leukot. Essent. Fatty Acids.

[26] Lee MJ, Chou FP, Tseng TH, Hsieh MH, Lin MC, Wang CJ (2002). Hibiscus protocatechuic acid or esculetin can inhibit oxidative LDL induced by either copper ion or nitric oxide donor. J. Agric. Food Chem.

[27] Holland JA, O'Donnell RW, Chang MM, Johnson DK, Ziegler LM (2000). Endothelial cell oxidant production: effect of NADPH oxidase inhibitors. Endothelium.

[28] Li X, Wang X, Chen D, Chen S (2011). Antioxidant activity and mechanism of protocatechuic acid in-vitro. Funct. Foods Health Dis.

[29] Virdis A, Bacca A, Colucci R, Duranti E, Fornai M, Materazzi G, Ippolito C, Bernardini N, Blandizzi C, Bernini G, Taddei S (2013). Endothelial dysfunction in small arteries of essential hypertensive patients: role of cyclooxygenase-2 in oxidative stress generation. Hypertension.

[30] Akpaffiong MJ, Taylor AA (1998). Antihypertensive and vasodilator actions of antioxidants in spontaneously hypertensive rats. Am. J. Hypertens.

[31] Vasdev S, Ford CA, Parai S, Longerich L, Gadag V (2001). Dietary vitamin C supplementation lowers blood pressure in spontaneously hypertensive rats. Mol. Cell. Biochem.

[32] Michel C, Cabanac M (1999). Effects of dexamethasone on the body weight set point of rats. Physiol. Behav.

[33] Lerario DDG, Ferreira SRG, Miranda WL, Chacra AR (2001). Influence of dexamethasone and weight loss on the regulation of serum leptin levels in obese individuals. Braz. J. Med. Biol. Res.

[34] Chmielewski V, Drupt F, Morfin R (2000). Dexamethasone-induced apoptosis of mouse thymocytes: Prevention by native 7α-hydroxysteroids. Immunol. Cell Biol.

[35] Kong F, Chen CH, Cooper MD (2002). Reversible disruption of thymic function by steroid treatment. J. Immunol.

